# Comparison of internal fixation outcomes in young femoral neck fractures: three cannulated compression screws versus the femoral neck system

**DOI:** 10.3389/fsurg.2026.1781680

**Published:** 2026-10-27

**Authors:** Ben Xiao, Meng Lu, Xiaobin Chen, Daojing Qiu, Yuanming He, Xuejun Li

**Affiliations:** Orthopedic Department of Beijing TongRen Hospital Affiliated Capital Medical University, Beijing, China

**Keywords:** cannulated compression screws, clinical effects, femoral neck fracture, femoral neck system, internal fixation

## Abstract

**Background:**

Femoral neck fractures in young patients typically result from traumatic injury, present significant treatment challenges, and are associated with a high risk of complications. The cannulated compression screw (CCS) is a common fixation device, whereas the femoral neck system (FNS) is a newer fixation device.

**Objective:**

To compare the clinical efficacy of the FNS and CCS in the treatment of femoral neck fractures in young patients.

**Methods:**

Between September 2021 and September 2023, 82 patients with femoral neck fracture aged 18–60 years were admitted to our hospital. According to the different fixation methods, the patients were divided into the FNS (38 cases) and CCS (44 cases) groups. The perioperative indicators, complications and follow-up function scores were compared between the two groups, and the follow-up time was ≥ 12 months.

**Results:**

The FNS group had shorter operation time (53.71 ± 11.20 vs. 64.61 ± 11.27 min, *p* < 0.001) and fewer intraoperative fluoroscopy times (14.05 ± 3.13 vs. 21.55 ± 5.06, *p* < 0.001). However, the incision length (38.05 ± 8.85 mm vs. 20.32 ± 6.34 mm, *p* < 0.001) and intraoperative blood loss (85.82 ± 23.62 mL vs. 65.89 ± 20.98 mL, *p* < 0.001) were greater. The FNS group demonstrated a lower rate of fixation failure (5.26% vs. 22.73%, *p* = 0.026) and a lower incidence of lateral thigh irritation (7.89% vs. 29.55%, *p* = 0.014), along with less severe femoral neck shortening (4.68 ± 2.04 vs. 7.14 ± 2.27 mm, *p* < 0.001). Patients in the FNS group achieved partial weight-bearing (3.08 ± 1.16 vs. 5.30 ± 1.50 weeks, *p* < 0.001) and full weight-bearing (11.71 ± 1.84 vs. 14.39 ± 2.07 weeks, *p* < 0.001) significantly earlier. The VAS scores at 3 and 7 days after surgery were significantly lower (*p* < 0.001), and the Harris scores at 3, 6, and 12 months after surgery were significantly higher (*p* < 0.05); however, the magnitude of Harris score differences fell below the minimal clinically important difference (MCID), and both groups achieved excellent final outcomes.

**Conclusion:**

Compared to CCS, FNS has better intraoperative efficiency, a lower internal fixation failure rate, and less severe femoral neck shortening in the treatment of femoral neck fractures in young adults. However, given the retrospective design, relatively small sample size, limited follow-up time, and differential follow-up duration between the groups, these conclusions should be interpreted with caution.

## 1 Introduction

Femoral neck fractures are the most common type of hip fracture, accounting for 48%–54% of all hip fractures (1–3). With population aging and rising traumatic events, femoral neck fracture incidence is increasing annually (4, 5). Although femoral neck fractures predominantly occur in the elderly population, with young patients accounting for approximately 3% of all hip fractures, these cases often present significant challenges. The cohort included injuries resulting from diverse mechanisms: 60% from traffic accidents, 17% from falls from heights, and 23% from falls on level ground. Such injuries frequently involve substantial displacement, large Pauwels angles, and poor fracture stability, significantly increasing treatment complexity (6–8). Postoperative femoral head necrosis in young patients can reach 10%–23%, with nonunion occurring in approximately 10%–30% of cases and a secondary surgery rate as high as 20%–48% (9–11). Therefore, for young patients, the treatment aims to achieve anatomical reduction and solid internal fixation to preserve blood supply to the femoral head to the greatest extent possible, reduce the risk of complications, and avoid or delay joint replacement (12).

Fixation with three cannulated compression screws (CCS) is the classic method for femoral neck fracture treatment and has been widely used in clinical practice because of its minimally invasive nature, simple operation, and low cost (13, 14). CCS promotes fracture healing via a sliding compression mechanism and reduces fracture-end micromovement, thereby improving the healing rate (15). However, CCS has significant limitations in treating displaced femoral neck fractures with poor intrinsic stability. Studies have shown that CCS performs poorly in terms of shear and rotational resistance, particularly in Pauwels type III fractures with a high rate of fixation failure (16, 17). In addition, the screw distribution of CCS requires precise operation, and multiple fluoroscopies to adjust the screw position not only increase the operation time and difficulty, but may also lead to bone loss and reduce the stability of internal fixation (18–20). Recently, the femoral neck system (FNS) has been introduced into clinical practice as a newer fixation device. FNS integrates a minimally invasive plate-screw construct comprising: (i) a locking plate contoured to the lateral femoral cortex, (ii) a central anti-rotation screw with a diameter of 10.0–11.5 mm that provides primary angular stability, and (iii) a sliding mechanism that permits controlled compression at the fracture site while maintaining rotational stability. The plate is fixed to the femoral shaft with two to three locking screws, forming an integrated load-bearing structure (21, 22). Biomechanical studies indicate that FNS outperforms traditional CCS in resisting shear and rotational forces and effectively reduces femoral neck shortening and femoral head necrosis (23). Additionally, the minimally invasive design of the FNS reduces surgical trauma and postoperative soft tissue irritation, thereby facilitating early patient recovery (24). Nevertheless, the clinical application of FNS is still in its developmental stage, and its long-term efficacy and cost-effectiveness require further validation. Although meta-analyses by Wu et al. (2022) (23), Zhou et al. (2023) (24), and Rajnish et al. (2022) (25) combined efficacy comparisons between FNS and CCS, these pooled analyses were limited by significant heterogeneity in patient demographics, fracture type, and surgical protocols among the included studies. Most previous studies focused on low-energy osteoporotic fractures in mixed-age or elderly populations, whereas femoral neck fractures are less common in younger adults. Furthermore, few available studies have provided detailed perioperative data, including precise fluoroscopic counts, standardized rehabilitation protocols with clear weight-bearing timelines, and digital measures of femoral neck shortening severity validated in a homogenous cohort of young patients using standardized surgical techniques. This study addresses this gap by performing a tightly controlled, single-center, retrospective analysis of young adults with femoral neck fractures, standardized surgical techniques performed by two experienced surgeons, and comprehensive perioperative and functional outcome assessments.

## 2 Materials and methods

### 2.1 General data

The clinical data of 124 young patients with femoral neck fractures treated at our hospital from September 2021 to September 2023 were retrospectively collected. A total of 42 patients were excluded per the pre-specified criteria. Ultimately, 82 patients were included in the study. According to the different fixation methods, the patients were divided into the FNS (38 cases) and CCS (44 cases) groups. A detailed algorithm for patient screening is presented in Figure 1. This study was approved by the Medical Ethics Committee of our hospital and all patients provided written informed consent.

**Figure 1:**
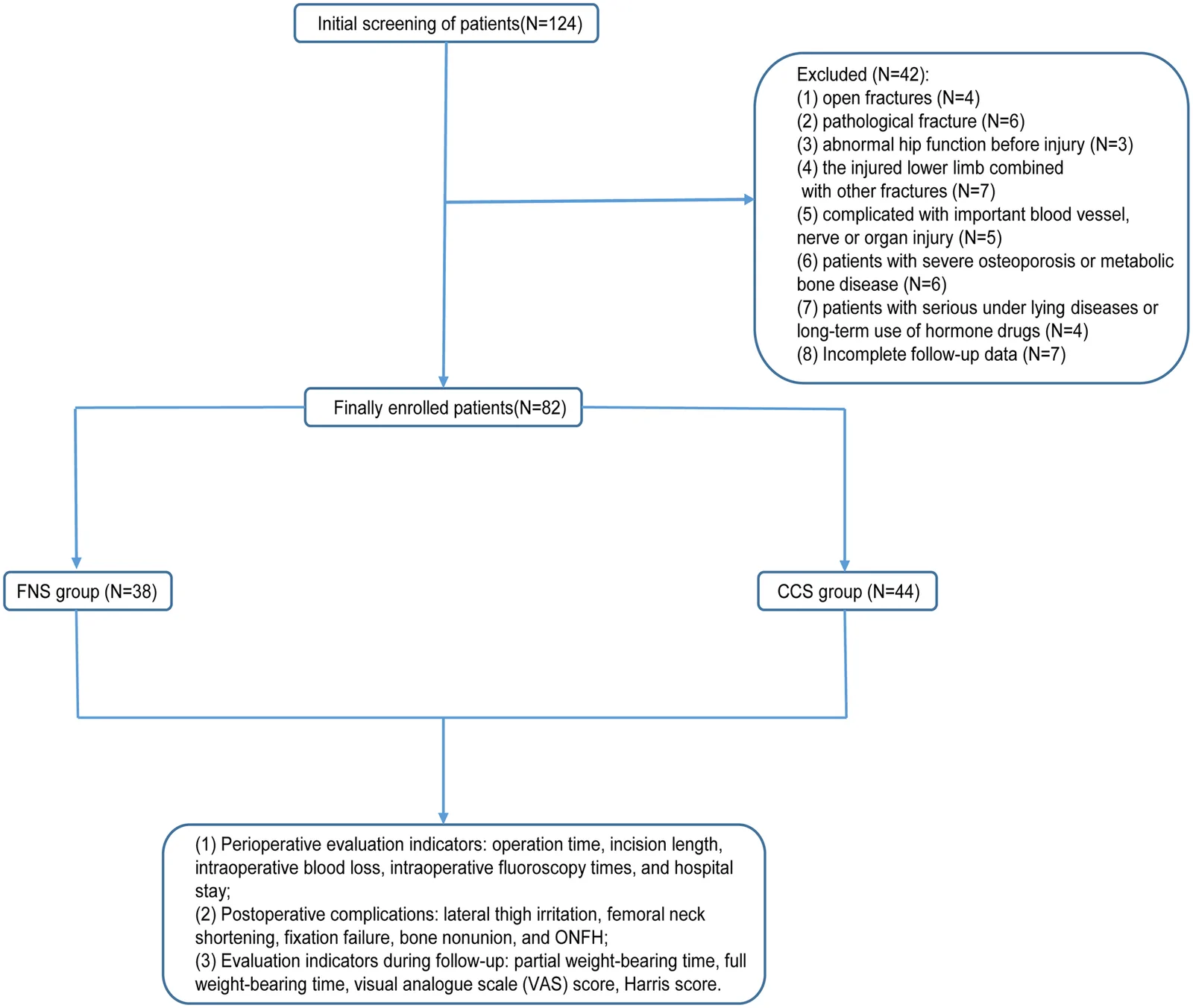
Flow chart of patient screening.

### 2.2 Inclusion and exclusion criteria

Inclusion criteria: (1) femoral neck fracture diagnosed by clinical and imaging; (2) fresh unilateral femoral neck fracture (operated within 3 weeks after injury); (3) age of 18–60 years; (4) internal fixation with FNS or CCS; (5) follow-up time ≥ 12 months; Exclusion criteria: (1) open fractures; (2) pathological fracture; (3) abnormal hip function before injury; (4) the injured lower limb combined with other fractures; (5) complicated with important blood vessel, nerve or organ injury; (6) patients with severe osteoporosis or metabolic bone disease; (7) patients with serious underlying diseases or long-term use of hormone drugs; (8) incomplete follow-up data. Notably, there were 26 Garden type II, 37 Garden type III, and 19 Garden type IV fractures among the patients in this study.

### 2.3 Preoperative preparation

After admission, the patient received routine treatment, including placing the affected limb in a neutral position for skin traction (weight 2–4 kg), subcutaneous injection of low-molecular-weight heparin (LMWH) sodium 5000 IU for anticoagulation therapy, and multimodal analgesia. Simultaneously, to improve the preoperative evaluation, all patients underwent three-dimensional hip CT reconstruction, lower limb color Doppler ultrasound, electrocardiogram, chest CT, and other routine examinations. In addition, prophylactic antibiotics were administered 30 min before surgery.

### 2.4 Surgical procedure

All surgeries were performed by two experienced orthopedic surgeons (an associate chief physician or a chief physician) in the Department of Orthopedics of our hospital to ensure that they were proficient in FNS and CCS fixation. Before this study, both surgeons had performed more than 100 procedures for each fixation technique (FNS and CCS), ensuring technical proficiency and consistency. The case distribution between the two surgeons was as follows: in the FNS group (*n* = 38), Surgeon A performed 18 surgeries (47.4%) and Surgeon B performed 20 surgeries (52.6%), whereas in the CCS group (*n* = 44), Surgeon A performed 21 surgeries (47.7%) and Surgeon B performed 23 surgeries (52.3%). There was no statistically significant difference in the distribution of cases between the two surgeons across the two groups (*χ*^2^ = 0.001, *P* = 0.974). Both surgeons performed FNS and CCS concurrently throughout the study period.

Implant selection criteria: Fixation method choice was guided by the fracture stability characteristics and Pauwels classification, referring to the institutional treatment protocol established in September 2021. FNS is generally preferred for Pauwels type III fractures (Pauwels angle >50°), displaced Garden III or IV fractures with poor intrinsic stability, and fracture patterns suggesting rotational instability. CCS was generally selected for Pauwels type I–II fractures (angle ≤50°) with relatively stable patterns and lower-displacement Garden II fractures, particularly when minimizing surgical trauma was prioritized. However, the final decision was made at the surgeon's discretion based on the intraoperative assessment of bone quality, fracture comminution, patient preference, and economic factors. Deviations from these guidelines have occurred in both directions. In the FNS group, six patients were treated in contrast to the guidelines: three due to intraoperative comminution not evident on preoperative imaging (including one case where good bone quality was also noted), two due to patient preference after an informed discussion of implant options, and one due to surgeon judgment based on the specific fracture pattern. In the CCS group, 10 patients were treated in contrast to the guidelines: four due to favorable bone quality or fracture geometry, three due to patient economic factors, two due to polytrauma considerations requiring minimal surgical time, and one due to patient preference.

In the FNS group, the patient was placed supine on an orthopedic traction table and received continuous epidural anesthesia combined with subarachnoid block anesthesia. The unaffected lower limb was positioned with hip flexion, knee flexion, and full abduction and was securely fixed using restraints. Under traction, closed reduction of the affected limb fracture was assessed by fluoroscopy using a C-arm x-ray machine. Satisfactory reduction was defined as anatomical or near-anatomical alignment (≤2 mm displacement, ≤5° angulation) with no posterior sagittal displacement on the lateral view. The Pauwels angle was measured on the anteroposterior (AP) view to confirm the fracture classification and verify that the reduction did not significantly alter the original fracture angulation. If the reduction was unsatisfactory, gentle manipulation (rotation, flexion/extension, and adduction/abduction) was performed under fluoroscopic guidance until acceptable alignment was achieved. To maintain the stability of the reduced fracture ends and prevent displacement during subsequent procedures, a 2.5 mm diameter Kirschner wire (K-wire) was first placed anterior to the femoral neck for temporary fixation. Subsequently, a longitudinal incision approximately 4 cm long was made along the femoral shaft axis. The skin and fascia were dissected layer-by-layer to expose the bone surface. A 2.5 mm diameter Steinmann guidewire was inserted in the midline direction of the femoral neck, with the tip advanced until it reached 5–10 mm from the subchondral bone of the femoral head. Subsequently, drilling and depth measurements were performed along the guide needle and a matching FNS nail plate system (Synthes, USA) was implanted. Bone plate position was confirmed using the anteroposterior and lateral fluoroscopic views via a C-arm x-ray machine, ensuring that the locking plate was centered on the lateral aspect of the femoral shaft. Locking screws and corresponding anti-rotation screws of appropriate length were sequentially inserted, and compression was applied until the fracture line disappeared. After confirming proper alignment and the absence of displacement at the fracture ends via fluoroscopy, and verifying satisfactory positioning of the internal fixation screws and osteosynthetic plates, the surgical field was cleaned using a pulsed irrigation device (Figures 2A–C).

**Figure 2:**
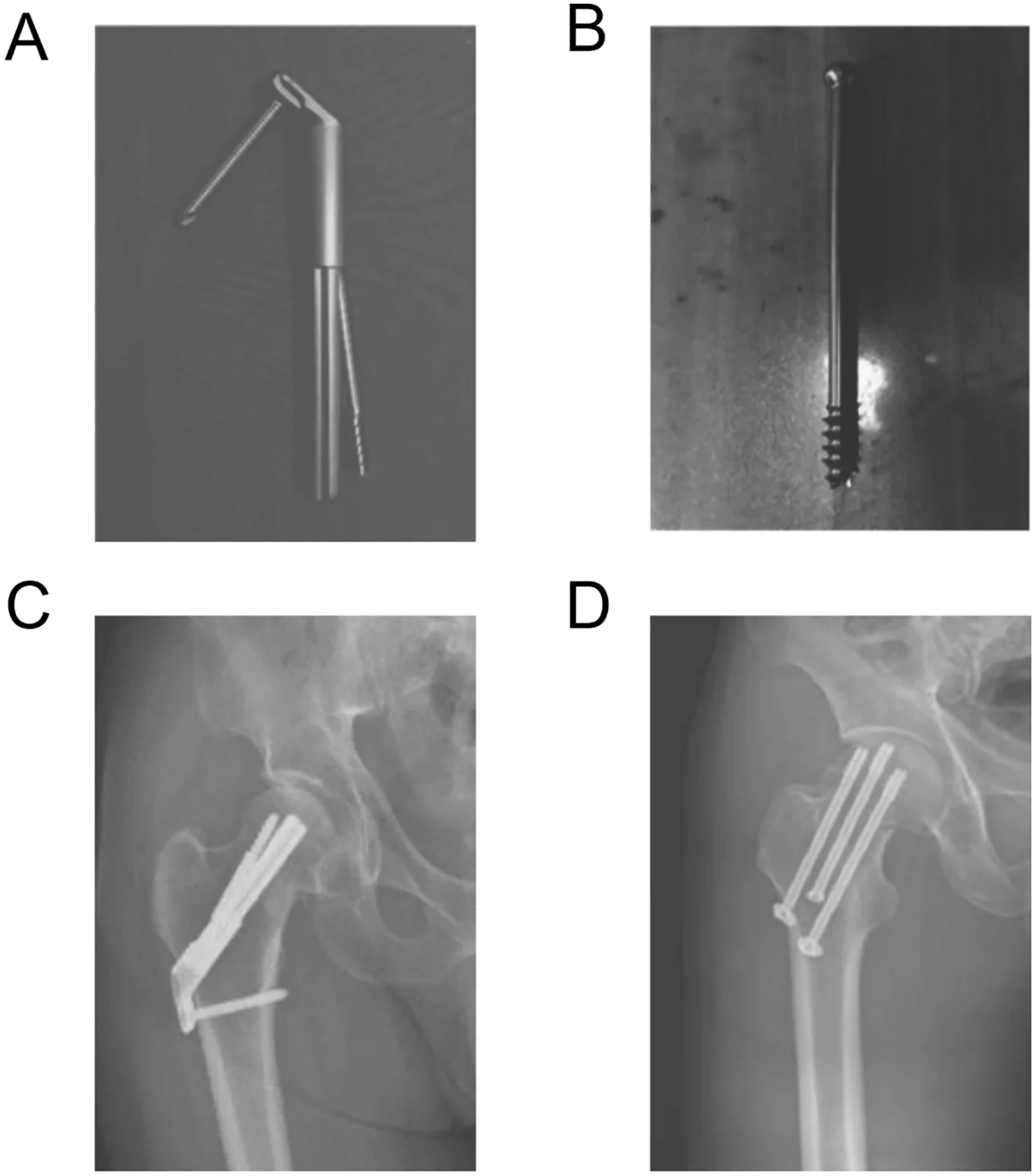
Two sets of fixation devices and anteroposterior and lateral x-ray images of a right femoral neck fracture. **(A)**: FNS fixation device. **(B)**: CCS fixation device. **(C)**: Three months postoperatively in the FNS group, the nail-plate system shows good positioning with blurred fracture lines. **(D)**: Three months postoperatively in the CCS group, the screws show good positioning with clear fracture lines. FNS, femoral neck system; CCS, cannulated compression screw.

CCS group: Anesthesia and patient positioning were identical to those in the FNS group. Following satisfactory reduction under C-arm fluoroscopy, the femoral neck was temporarily stabilized with 2.5 mm K-wires using the same technique. Subsequently, the skin and fascia on the bone surface were incised. Under preoperative fluoroscopic guidance, three parallel CCS screws (Synthes, USA) were inserted into the femoral neck through predefined entry points. C-arm fluoroscopy was used to visualize the hip in the anteroposterior and lateral views. After confirming satisfactory fracture reduction and optimal fixation, the incision was irrigated using a pulsed irrigation device (Figures 2B–D).

### 2.5 Postoperative care

Postoperatively, all patients received routine prophylactic antibiotic therapy and were administered oral or intravenous analgesics, as needed, based on their pain levels. Anteroposterior and lateral x-rays of the affected hip were reviewed on the second postoperative day. Rehabilitation followed a standardized, three-phase protocol identical for both groups. The initial phase (postoperative weeks 1–2) focused on isometric contractions and passive range-of-motion exercises. The intermediate phase (postoperative week 3 until radiographic evidence of fracture healing) transitioned to non-weight-bearing joint mobilization of the lower limbs and muscle strength training for muscle groups, such as the quadriceps. The final phase involved progressive weight-bearing joint function, muscle strength enhancement, and balance training. Progression to partial and full weight-bearing was determined uniformly for both groups based on standardized radiographic and clinical criteria, including (i) visible callus formation or fracture line blurring on anteroposterior and lateral hip radiographs; (ii) absence of pain at the fracture site during hip range-of-motion testing; and (iii) no radiographic evidence of fixation failure, screw cut-out, or displacement. The attending surgeons consistently applied these criteria. This decision was not solely based on patient comfort. Blinding to the implant type is not feasible because the FNS and CCS structures are easily distinguished on plain radiographs. Key rehabilitation exercise points were thoroughly explained to the patient and family, with instructions to continue training after discharge. Follow-up visits were required at 1, 2, 3, and 6 months after discharge.

### 2.6 Observational index

(1) Perioperative evaluation indicators: operation time, incision length, intraoperative blood loss, intraoperative fluoroscopy times, and hospital stay. Intraoperative blood loss was estimated by combining (i) blood volume in suction canisters (minus irrigation fluid); (ii) blood absorbed by gauzes (10 mL per fully soaked 4 × 4-inch gauze, 100 mL per laparotomy sponge); and (iii) visual estimation of blood in surgical fields. The total was recorded in the anesthesia and surgical notes by both the anesthesiologist and the lead surgeon, with discrepancies >20% resolved by consensus. (2) Postoperative complications: lateral thigh irritation, femoral neck shortening, fixation failure, bone nonunion, and ONFH, with the following standardized definitions and assessment protocols: Fixation failure was defined radiographically as any of the following: (i) loss of reduction (>3 mm displacement or >10° angulation compared with immediate postoperative films); (ii) varus collapse (decrease in neck-shaft angle >10°); (iii) implant cut-out or back-out (>5 mm migration or joint penetration); or (iv) implant breakage. Nonunion was defined as the absence of radiographic healing at 6 months postoperatively, characterized by persistent fracture lines with sclerotic margins and no bridging calluses on the three cortices. Assessment time points: 3, 6, and 12 months. ONFH was diagnosed using the Ficat-Arlet staging system based on plain radiographs and MRI, when indicated as Stage I (MRI-positive only), Stage II (cystic/sclerotic changes), Stage III (subchondral collapse/crescent sign), or Stage IV (joint space narrowing). Assessment time points were 6 and 12 months; MRI was performed for equivocal radiographic findings. Femoral neck shortening length was measured on standard anteroposterior hip radiographs taken 2 days postoperatively and at the last follow-up using the hospital PACS digital measurement tool. The measurement was performed along a vertical line from the center of the femoral head (rotation center) to the intersection point with the inferior border of the femoral neck (the medial cortex at the base of the femoral neck, where it transitions to the intertrochanteric line). Shortened length was defined as the difference between the postoperative and last follow-up measurements. Femoral neck shortening was classified as none/slight (<5 mm), moderate (5–10 mm), or severe (>10 mm), based on the absolute shortening length. Two independent orthopedic surgeons performed the measurements, and the mean values were used for statistical analysis. (3) Evaluation indicators during follow-up included partial weight-bearing time, full weight-bearing time, visual analog scale (VAS) score, and Harris score.

### 2.7 Statistical analysis

SPSS 22.0 and GraphPad Prism 8.0 software were used for analysis and mapping. Normally distributed measurement data were expressed as mean ± standard deviation and compared using an independent sample t-test. Non-normally distributed data were expressed as median (interquartile range) and compared using the Mann–Whitney U test. Count data were expressed as [n (%)]. For comparisons of categorical variables with expected cell counts ≥5, the chi-square test without continuity correction was used, as Yates’ correction is overly conservative with small samples and unequal group sizes. Fisher's exact test was used for comparisons with expected cell counts <5. Given the retrospective design and limited number of fixation failure events (*n* = 12), Firth penalized logistic regression was performed for the primary outcome to reduce small-sample bias. Firth's penalized logistic regression was performed using R (version 4.2.1; R Foundation for Statistical Computing, Vienna, Austria) with the logistf package (version 1.24), which implements this method via a penalized maximum likelihood with a Jeffreys prior penalty. Kaplan–Meier survival analysis and log-rank test were performed using R (version 4.2.1; R Foundation for Statistical Computing, Vienna, Austria) with the survival package (version 3.4–0) and Survminer package (version 0.4.9). Two parsimonious models used only pre-operative covariates: Model 1 (primary adjusted analysis) was adjusted for Pauwels classification only, and Model 2 (sensitivity analysis) was adjusted for age, BMI, Garden classification, Pauwels classification, and time from injury to surgery. Femoral neck shortening severity was excluded, as it is a post-operative mediator rather than a confounder. Adjusted odds ratios (aOR) and 95% confidence intervals (CI) were calculated. *P* < 0.05 was considered statistically significant.

## 3 Results

### 3.1 General data

A total of 82 young patients with femoral neck fractures were included in this study. There were 38 patients in the FNS group, including 22 men and 16 women, aged (40.32 ± 7.72) years. There were 44 patients in the CCS group, including 26 men and 18 women, aged (43.14 ± 7.66) years. There were no significant differences in the preoperative general data, such as age, sex, BMI, time from injury to operation, side, Garden classification, Pauwels classification, and cause of injury between the two groups (*P* > 0.05) (Table 1).

**Table 1:** Comparison of general information of the two groups of patients.

Classification	FNS group (*n* = 38)	CCS group (*n* = 44)	t/*χ*2	*P* value
Gender			0.012	0.913
Male	22 (57.89)	26 (59.09)		
Female	16 (42.11)	18 (40.91)		
Age	40.32 ± 7.72	43.14 ± 7.66	1.657	0.102
BMI	23.80 ± 2.13	23.39 ± 2.21	0.866	0.389
Time from injury to surgery	2.76 ± 1.10	2.60 ± 1.01	0.710	0.480
Side distinction			0.030	0.862
Left	20 (52.63)	24 (54.55)		
Right	18 (47.37)	20 (45.45)		
Garden typing			0.446	0.800
II	12 (31.58)	14 (31.82)		
III	16 (42.10)	21 (47.73)		
IV	10 (26.32)	9 (20.45)		
Pauwels angle			0.455	0.796
< 30°	15 (39.47)	18 (40.91)		
30°-50°	12 (31.58)	16 (36.36)		
> 50°	11 (28.95)	10 (22.73)		
Cause of injury			0.083	0.959
Traffic accident	23 (60.53)	26 (59.09)		
Fall from height	6 (15.79)	8 (18.18)		
Falling on level ground	9 (23.68)	10 (22.73)		
Surgeon			0.001	0.974
Surgeon A	18 (47.37)	21 (47.73)		
Surgeon B	20 (52.63)	23 (52.27)		

FNS, femoral neck system; CCS, cannulated compression screw; BMI, body mass index. No Garden I fractures were identified in this cohort.

### 3.2 Perioperative

All patients in both groups completed surgery without serious complications. In the FNS group, the surgery time (53.71 ± 11.20 min vs. 64.61 ± 11.27 min, *p* < 0.001) and the number of intraoperative fluoroscopy (14.05 ± 3.13 times vs. 21.55 ± 5.06 times, *p* < 0.001) were significantly less than the CCS group. The FNS group had significantly longer incisions (38.05 ± 8.85 mm vs. 20.32 ± 6.34 mm, *p* < 0.001) and intraoperative blood loss (85.82 ± 23.62 mL vs. 65.89 ± 20.98 mL, *p* < 0.001) compared to the CCS group. There was no statistically significant difference in hospital stay between the two groups (11.61 ± 1.48 days vs. 12.05 ± 1.26 days, *p* = 0.149) (Table 2).

**Table 2:** Comparison of perioperative indicators between the two patient groups.

Index	FNS group (*n* = 38)	CCS group (*n* = 44)	t	*P* value
Surgery time (min, x̅ ± s)	53.71 ± 11.20	64.61 ± 11.27	4.382	<0.001
Intraoperative blood loss (mL, x̅ ± s)	85.82 ± 23.62	65.89 ± 20.98	4.046	<0.001
Number of intraoperative fluoroscopy procedures (time, x̅ ± s)	14.05 ± 3.13	21.55 ± 5.06	7.912	<0.001
Total incision length (mm)	38.05 ± 8.85	20.32 ± 6.34	10.53	<0.001
Length of hospital stay (d)	11.61 ± 1.48	12.05 ± 1.26	1.457	0.149

FNS, femoral neck system; CCS, cannulated compression screw.

### 3.3 Postoperative complications

Femoral neck shortening in the FNS group was significantly smaller than that in the CCS group (4.68 ± 2.04 mm vs. 7.14 ± 2.27 mm, *p* < 0.001). During follow-up, three cases (7.89%) in the FNS group and 13 cases (29.55%) in the CCS group developed soft tissue irritation at the implant site on the affected side, and the difference between the two groups was statistically significant (*P* = 0.014; risk difference = 0.217, 95% CI: 0.057–0.376). The fixation failure incidence was significantly higher in the CCS group than in the FNS group (10 [22.73%] vs. 2 [5.26%], *P* = 0.026; risk difference = 0.175, 95% CI: 0.032–0.317). In addition, nonunion occurred in seven patients, including two (5.26%) in the FNS group and five (11.36%) in the CCS group. Ten patients developed ONFH, including three (7.89%) in the FNS group and seven (15.91%) in the CCS group. No significant differences were observed in the nonunion (5.26% vs. 11.36%, *P* = 0.442) or ONFH (7.89% vs. 15.91%, *P* = 0.326) rates between the groups. However, based on published event rates, detecting a clinically meaningful difference would require approximately 280 patients (140 per group) for nonunion and 514 for ONFH, both at 80% power and *α* = 0.05. Our sample size of 82 patients was substantially underpowered for these secondary outcomes and the absence of statistically significant differences should not be interpreted as evidence of equivalence (Table 3).

**Table 3:** Comparison of postoperative complications between the two groups.

Classification	FNS group (*n* = 38)	CCS group (*n* = 44)	t/χ2	*P* value	RD (95% CI)
Shortened femoral neck (n, %)			1.715	0.424	
None or slight	21 (55.26)	18 (40.91)			
Moderate	12 (31.58)	19 (43.18)			
Severe	5 (13.16)	7 (15.91)			
Shortening length of the femoral neck (mm, x̅ ± s)	4.68 ± 2.04	7.14 ± 2.27	5.112	<0.001	
Lateral thigh irritation	3 (7.89)	13 (29.55)	6.086	0.014	0.217 (0.057–0.376)
Failure of internal fixation	2 (5.26)	10 (22.73)	4.978	0.026	0.175 (0.032–0.317)
Nonunion	2 (5.26)	5 (11.36)		0.442	
ONFH	3 (7.89)	7 (15.91)		0.326	

FNS, femoral neck system; CCS, cannulated compression screw; ONFH, osteonecrosis of the femoral head; Shortening severity was classified as: none or slight (< 5 mm), moderate (5–10 mm), and severe (> 10 mm). fixation failure, nonunion, and ONFH were recorded as mutually exclusive primary complications for each patient. If a patient developed multiple complications, classification was based on chronological order of diagnosis; if diagnosed simultaneously, the following severity priority was applied: fixation failure > nonunion > ONFH. Lateral thigh irritation was recorded as an independent complication and could coexist with the primary complications. The chi-square test without continuity correction was used for fixation failure and lateral thigh irritation, as Yates’ correction is overly conservative for small samples. Risk differences (RD) with 95% confidence intervals are reported alongside *p*-values; Fisher's exact test was used for nonunion and ONFH.

### 3.4 Multivariable analysis of fixation failure

Firth penalized logistic regression was performed for fixation failure using only pre-operative covariates. In the primary adjusted analysis (Model 1), FNS fixation was associated with a significantly lower risk of fixation failure compared to CCS (adjusted OR=0.20, 95% CI: 0.05–0.91, *P* = 0.037). In the sensitivity analysis (Model 2), the protective effect of FNS persisted (adjusted OR=0.22, 95% CI: 0.05–0.98, *P* = 0.047), although the wide confidence intervals reflect the limited number of events (*n* = 12) (Table 4).

**Table 4:** Firth penalized logistic regression analysis of factors associated with fixation failure .

Variable	Model	Adjusted OR	95% CI	*P* value
Fixation method (FNS vs CCS)	Model 1	0.20	0.05–0.91	0.037
Pauwels angle (>50° vs <30°)	Model 1	2.90	0.78–10.81	0.113
Fixation method (FNS vs CCS)	Model 2	0.22	0.05–0.98	0.047
Age	Model 2	1.06	0.97–1.15	0.189
BMI	Model 2	0.85	0.64–1.12	0.247
Garden classification (III vs II)	Model 2	4.07	0.35–46.84	0.260
Garden classification (IV vs II)	Model 2	1.49	0.03–76.71	0.843
Pauwels angle (30°–50° vs <30°)	Model 2	0.63	0.07–5.57	0.675
Pauwels angle (>50° vs <30°)	Model 2	2.91	0.09–93.14	0.545
Time from injury to surgery	Model 2	0.87	0.47–1.63	0.668

OR, odds ratio; CI, confidence interval; FNS, femoral neck system; CCS, cannulated compression screw; BMI, body mass index. Model 1 (primary adjusted analysis) adjusted for Pauwels classification only. Model 2 (sensitivity analysis) adjusted for age, BMI, Garden classification, Pauwels classification, and time from injury to surgery.

### 3.5 Follow up

Both patient groups were followed up for 12 months or longer postoperatively. Mean follow-up duration was significantly shorter in the FNS group than in the CCS group (16.58 ± 3.52 vs. 18.28 ± 3.12 months, *p* = 0.023). Figure 3 shows the Kaplan–Meier survival curves for the composite end point of internal fixation failure, nonunion, or osteonecrosis of the femoral head. The event-free survival rate of the FNS group was better than that of the CCS group (log-rank test, *χ*^2^ = 7.12, *P* = 0.008). Patients in the FNS group achieved partial weight-bearing significantly earlier than those in the CCS group (3.08 ± 1.16 weeks vs. 5.30 ± 1.50 weeks, *p* < 0.001) and full weight-bearing significantly earlier (11.71 ± 1.84 weeks vs. 14.39 ± 2.07 weeks, *p* < 0.001) (Figures 4A,B). Both groups showed decreasing VAS scores over time postoperatively. The FNS group had significantly lower VAS scores than the CCS group on postoperative day 3 (3.42 ± 1.20 vs. 4.46 ± 1.44, *p* < 0.001) and day 7 (2.47 ± 1.08 vs. 3.75 ± 1.10, *p* < 0.001) (Figure 4C). Both groups showed increasing Harris scores over time postoperatively. The FNS group showed significantly higher scores at 3 months (72.97 ± 6.03 vs. 70.09 ± 6.26, *p* = 0.037), 6 months (91.82 ± 4.57 vs. 88.71 ± 4.25, *p* = 0.002), and 12 months (94.58 ± 3.16 vs. 91.11 ± 3.27, *p* < 0.001) than the CCS group (Figure 4D).

**Figure 3:**
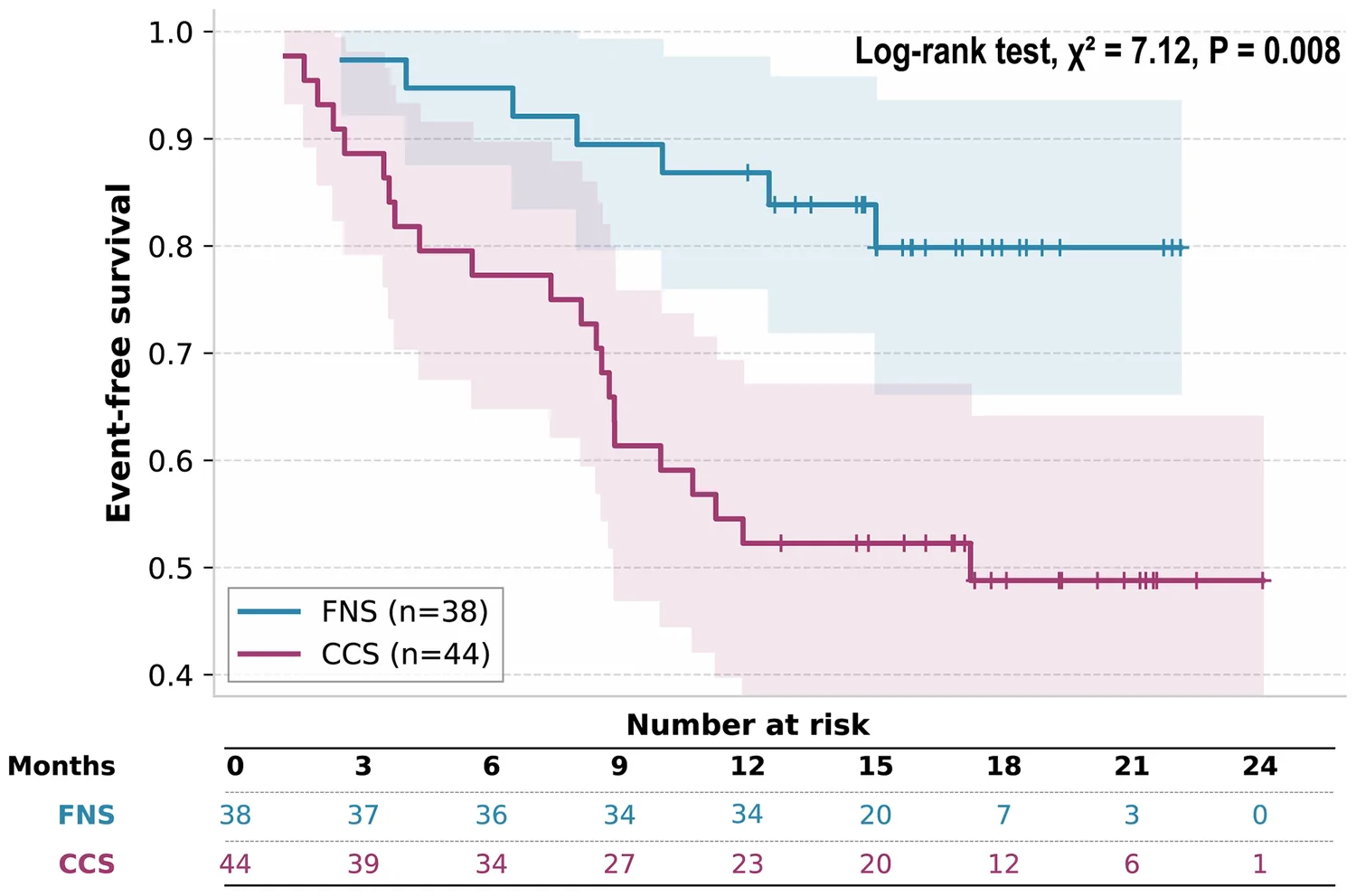
Kaplan–Meier survival curves for the composite endpoint of fixation failure, nonunion, or osteonecrosis of the femoral head. Patients were censored at the last follow-up. The FNS group demonstrated significantly better event-free survival compared with the CCS group (log-rank test, *χ*^2^ = 7.12, *P* = 0.008). Tick marks indicate censored observations. Composite events were defined as the first occurrence of fixation failure, nonunion, or ONFH. Patients with multiple complications contributed only their first event in chronological order to the survival analysis.

**Figure 4:**
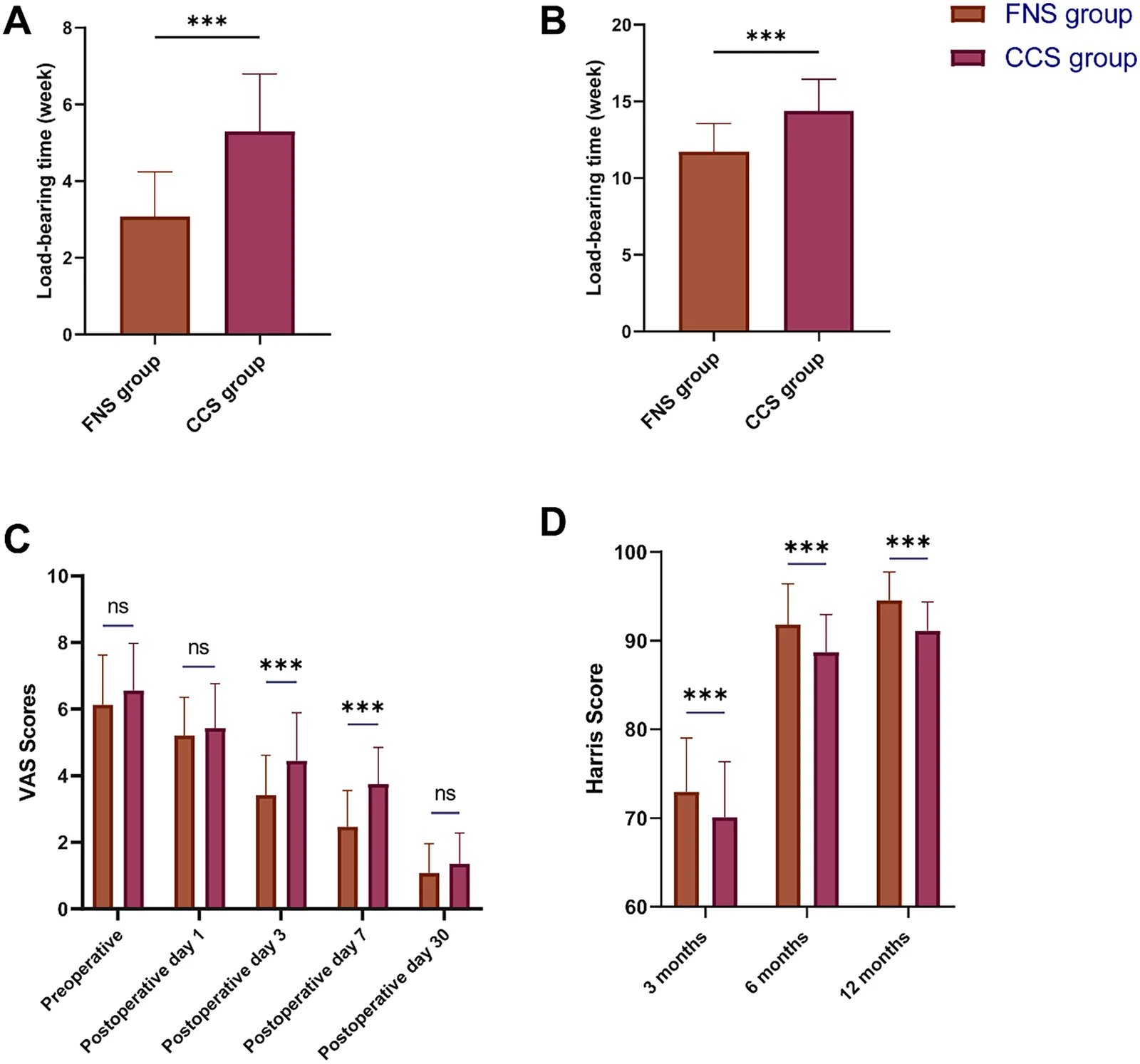
Comparison of follow-up data. **(A)**: Partial weight-bearing time after surgery. **(B)**: Full weight-bearing time after surgery. **(C)**: VAS scores before surgery and on postoperative days 1, 3, 7, and 30. **(D)**: Harris scores at 3, 6, and 12 months after surgery. VAS, visual analogue scale.

## 4 Discussion

This study aimed to compare the clinical efficacy of the femoral neck system (FNS) and three CCS for the treatment of femoral neck fractures in young patients. The results showed that the FNS group outperformed the CCS group in shorter operation time, fewer intraoperative fluoroscopy times, lower fixation failure rate, less severe femoral neck shortening, earlier achievement of partial and full weight-bearing, lower early postoperative VAS scores, and higher Harris hip scores at 3, 6, and 12 months. Although the incision length and intraoperative blood loss were greater in the FNS group, postoperative recovery and hospital stay were not significantly affected. These findings support the clinical advantages of FNS for treating femoral neck fractures in young adults and a reference for individualized treatment.

The operation and intraoperative fluoroscopy times in the FNS group were significantly shorter than those in the CCS group, which were related to the systematic design of the FNS and the built-in guidance device. FNS uses an integrated screw-plate structure, eliminating repeated screw-position adjustments and reducing the number of surgical steps and the need for fluoroscopy, thereby shortening the operation time and reducing the radiation exposure of both doctors and patients (26). In contrast, although CCS is a classic minimally invasive surgery, its screws require repeated fluoroscopic adjustment, especially in high-angle Pauwels fractures that are difficult to operate, and fluoroscopy counts naturally increase. Meta-analyses by Wu et al. (2022) (23) and Zhou et al. (2023) (24) reported wide confidence intervals for procedure times owing to heterogeneity in the included surgeons’ experience and institutional protocols. In contrast, the standardized surgical protocol used in this study produced consistent and precise reductions in operative and fluoroscopic times, reinforcing the internal validity of these findings in young adults with femoral neck fractures. The anti-shear and anti-rotational abilities of CCS are weak, especially in Pauwels type III fractures, which are prone to screw cutting, backsliding, or breakage, leading to internal fixation failure (27, 28). Rajnish et al. (2022) (25) reported that FNS was associated with a lower rate of fixation failure than CCS, but noted significant heterogeneity across studies. This study demonstrated a fixation failure rate of 5.26% in the FNS group versus 22.73% in the CCS group in young adults with femoral neck fractures (*p* = 0.026), within Rajnish et al.'s reported range, but with the advantage of a homogeneous patient population and a standardized surgical protocol. Through dynamic compression and a locking plate structure, the FNS forms a more stable mechanical system that can effectively resist vertical shear and rotational stress and reduce the risk of fixation failure (29, 30). In the primary adjusted analysis (Model 1), FNS fixation remained associated with a lower risk of fixation failure (aOR = 0.20, 95% CI: 0.05–0.91, *P* = 0.037). In the sensitivity analysis (Model 2), the association persisted (aOR = 0.22, 95% CI: 0.05–0.98, *P* = 0.047), although the wide confidence intervals reflect the limited number of events (*n* = 12) and the estimate should be interpreted with caution. This finding suggests an association between FNS fixation and lower fixation failure rates, consistent with the biomechanical advantages of the FNS construct. However, in this non-randomized study with a small number of events, protocol deviations, and unmeasured confounders such as reduction quality, residual confounding could not be distinguished from a genuine implant effect, and the adjusted estimates should be interpreted with caution. Although the FNS group had statistically greater intraoperative blood loss than the CCS group (85.82 ± 23.62 mL vs. 65.89 ± 20.98 mL, *p* < 0.001), the absolute difference is clinically negligible and unlikely to impact perioperative hemodynamic stability or transfusion requirements. This finding should be interpreted as a reflection of the more extensive soft tissue dissection required for plate insertion rather than a clinically meaningful disadvantage. Soft tissue irritation was significantly less common in the FNS group than in the CCS group (7.89% vs. 29.55%, *p* = 0.014), facilitating early functional exercises. Femoral neck shortening is an important factor that affects hip joint function, especially in young patients. Preservation of the femoral neck length significantly delays joint degeneration and avoids early joint replacement (31, 32). Femoral neck shortening was significantly smaller in the FNS group than in the CCS group, indicating that FNS better maintained the stability of the fracture end and prevented collapse. The sliding compression mechanism of the FNS can effectively control excessive shortening while promoting fracture healing, which is related to the good axial stability and anti-compression ability of the system (33). The Harris Hip Scores were significantly higher in the FNS group at 3, 6, and 12 months; however, these differences fell below the commonly accepted minimal clinically important difference (MCID) of 8–10 points for the HHS. Both groups achieved excellent final outcomes (>90 points at 12 months), suggesting that any marginal early functional advantage from FNS should be interpreted with caution. It remains uncertain whether this statistically significant sub-MCID improvement translates into meaningful, long-term functional benefits. Similarly, the VAS scores were lower in the FNS group 3 and 7 days after surgery, and no between-group differences were observed 30 days after surgery. Thus, although the FNS may be associated with a slight reduction in early postoperative discomfort, this finding should not be overinterpreted as a definitive clinical advantage. Early restoration of weight bearing and joint function is essential in young patients with femoral neck fracture (25, 34). The absence of significant differences in nonunion and ONFH between the FNS and CCS groups should be interpreted with caution. Mean follow-up duration was significantly longer in the CCS group (18.28 ± 3.12 vs. 16.58 ± 3.52 months, *P* = 0.023); as these are time-dependent outcomes, the longer observation period may have contributed to higher observed complication rates in the CCS group. Moreover, our sample size was substantially underpowered for these secondary outcomes and the absence of statistically significant differences should not be interpreted as evidence of equivalence. The times for partial and full weight-bearing in the FNS group were earlier than those in the CCS group. While these differences were statistically significant, the clinical importance of earlier weight-bearing should be interpreted cautiously given the absence of corresponding differences in hospital stay and the sub-MCID nature of functional score improvements. These findings should be considered in the context of broader treatment goals for femoral neck fractures in young patients. The most important clinical outcome in this study was not small differences in early pain scores or sub-MCID changes in functional scores, but rather avoidance of internal fixation failure, nonunion, and secondary conversion to total hip arthroplasty, events that confer substantial morbidity and socioeconomic burden. The fixation failure rate was significantly lower in the FNS group than in the CCS group (RD = 0.175, 95% CI: 0.032–0.317). Although the lower confidence limit approached zero, indicating that the estimate was somewhat fragile, the point estimate suggested a clinically relevant reduction in fixation failure in the FNS group.

### 4.1 Limitations

This study had certain limitations. The single-center retrospective design may have introduced selection bias; implant selection deviated from the institutional protocol in 16 of 82 patients (19.5%): 6 in the FNS group and 10 in the CCS group. While the reasons for deviation were documented (intraoperative findings, patient preference, and economic factors), this reflects the design's inherent limitations and may introduce residual confounding despite multivariable adjustment. Additionally, the hierarchical complication classification (fixation failure > nonunion > ONFH) differentially suppressed the recorded nonunion counts in the CCS group because of the higher fixation failure rate, which biased the nonunion comparison toward the null. Therefore, the true nonunion rate in the CCS group may have been higher than that observed. The outcome assessment was not blinded to the implant type, as the FNS and CCS constructs are readily distinguishable on plain radiographs, representing a potential source of detection bias. The sample size was relatively small, particularly limiting the statistical power for nonunion and ONFH comparisons; approximately 280 and 514 patients, respectively, would be required to detect clinically meaningful differences in these outcomes. Mean follow-up duration was significantly longer in the CCS group than in the FNS group; as nonunion and ONFH are time-dependent outcomes, the longer observation period may have contributed to higher observed complication rates in the CCS group. The follow-up period was short, preventing the assessment of long-term complications such as ONFH, which typically becomes evident 2–3 years after injury. Therefore, the reported incidence of ONFH should be considered preliminary. Several important prognostic factors have not been reported or controlled for, including the quality of fracture reduction (Garden index), number of intraoperative reduction attempts, and bone mineral density. Although the baseline fracture characteristics were comparable between the groups, unmeasured differences in reduction quality may partially explain the observed differences in complications. Furthermore, no cost-effectiveness analysis was conducted, and although FNS is more expensive than CCS, its long-term economic benefits warrant further investigation. Larger, prospective, multi-center randomized trials with longer follow-up are needed to verify the long-term effects of FNS.

## 5 Conclusion

Compared with CCS, FNS has better intraoperative efficiency, a lower internal fixation failure rate, and less severe femoral neck shortening for treating femoral neck fractures in young adults. However, given the retrospective design, limited sample size, and short follow-up period, these results should be considered preliminary. Multicenter, prospective randomized trials and long-term follow-up studies are needed to verify its efficacy and safety and to explore its application value in elderly patients and special fracture types.

## Data Availability

The raw data supporting the conclusions of this article will be made available by the authors, without undue reservation.

## References

[1] Collinge CA Finlay A Rodriguez-Buitrago A Beltran MJ Mitchell PM Mir HR. Treatment failure in femoral neck fractures in adults less than 50 years of age: analysis of 492 patients repaired at 26 north American trauma centers. J Orthop Trauma. (2022) 36:271–9. 10.1097/bot.0000000000002355 35703846

[2] Bhandari M Swiontkowski M. Management of acute hip fracture. N Engl J Med. (2017) 377:2053–62. 10.1056/NEJMcp1611090 29166235

[3] Ye Y Hao J Mauffrey C Hammerberg EM Stahel PF Hak DJ. Optimizing stability in femoral neck fracture fixation. Orthopedics. (2015) 38:625–30. 10.3928/01477447-20151002-05 26488776

[4] Wu A-M Bisignano C James SL Abady GG Abedi A Abu-Gharbieh E. Lobal, regional, and national burden of bone fractures in 204 countries and territories, 1990−2019: a systematic analysis from the global burden of disease study 2019. The Lancet. Healthy Longevity. (2021) 2:e580–92. 10.1016/s2666-7568(21)00172-0 34723233 PMC8547262

[5] Chen W Lv H Liu S Liu B Zhu Y Chen X. National incidence of traumatic fractures in China: a retrospective survey of 512 187 individuals. The Lancet. Global Health. (2017) 5:e807–17. 10.1016/s2214-109x(17)30222-x 28666814

[6] Hoskins W Rayner J Sheehy R Claireaux H Bingham R Santos R. The effect of patient, fracture and surgery on outcomes of high energy neck of femur fractures in patients aged 15–50. Hip Int. (2019) 29:77–82. 10.1177/1120700018761154 29734844

[7] Enocson A Lapidus LJ. The vertical hip fracture—a treatment challenge. A cohort study with an up to 9 year follow-up of 137 consecutive hips treated with sliding hip screw and antirotation screw. BMC Musculoskelet Disord. (2012) 13:171. 10.1186/1471-2474-13-171 22971243 PMC3495198

[8] Jin Y Yin B Shu L Fan Z Sherrier MC Liu C. Morphological characteristics of femoral neck fractures in young and middle-aged population: a retrospective descriptive study. BMC Musculoskelet Disord. (2024) 25:100. 10.1186/s12891-024-07207-5 38287282 PMC10823608

[9] Slobogean GP Sprague SA Scott T Bhandari M. Complications following young femoral neck fractures. Injury. (2015) 46:484–91. 10.1016/j.injury.2014.10.010 25480307

[10] Obey MR Falgons CG Eastman JG Choo AM Achor TS Munz JW. Low reoperation rate after fixation of displaced femoral neck fractures with the femoral neck system (FNS). European Journal of Orthopaedic Surgery & Traumatology: Orthopedie Traumatologie. (2024) 34:2581–8. 10.1007/s00590-024-03962-2 38700518

[11] Stockton DJ O’Hara LM O’Hara NN Lefaivre KA O’Brien PJ Slobogean GP. High rate of reoperation and conversion to total hip arthroplasty after internal fixation of young femoral neck fractures: a population-based study of 796 patients. Acta Orthop. (2019) 90:21–5. 10.1080/17453674.2018.1558380 30712497 PMC6366467

[12] Slobogean GP Sprague SA Scott T McKee M Bhandari M. Management of young femoral neck fractures: is there a consensus? Injury. (2015) 46:435–40. 10.1016/j.injury.2014.11.028 25530411

[13] Yuan K- Yang F Fu K Zhu D- Jiang C- Jin D- Internal fixation using fully threaded cannulated compression screws for fresh femoral neck fractures in adults. J Orthop Surg Res. (2022) 17:108. 10.1186/s13018-022-03005-8 35184732 PMC8859893

[14] Wang P Zhang Z Zhao Z Li Z Liu L Zhao K. Long-term outcomes of triple cannulated compression screws combined with bone graft sleeve parallel implantation of DBM crunch internal fixation for the treatment of femoral neck fractures in middle-aged and young adults. Orthop Surg. (2025) 17:3211–21. 10.1111/os.70169 41065639 PMC12580288

[15] Ji R Lu X Cong R Pan D. [Comparison of full thread compression cannulated screw and partial thread cannulated screw in treatment of femoral neck fracture]. Chin J Reparative Reconstr Surg. (2023) 37:19–24. 10.7507/1002-1892.202208103 PMC9883638 36708110

[16] Tianye L Peng Y Jingli X QiuShi W GuangQuan Z Wei H. Finite element analysis of different internal fixation methods for the treatment of pauwels type III femoral neck fracture. Biomed Pharmacother. (2019) 112:108658. 10.1016/j.biopha.2019.108658 30970508

[17] Wang J Gao Y Song D Ni J. Biomechanical study on parallel cannulated compression screw combined with medial buttress plate fixation and F-type cannulated compression screw fixation in pauwels III femoral neck fracture: a finite element analysis. Journal of Central South University. Medical Sciences. (2022) 47:1143–53. 10.11817/j.issn.1672-7347.2022.220022 PMC10950106 36097783

[18] Sun H Shu L-Y Sherrier MC Zhu Y Liu J-W Zhang W. Decreased complications but a distinctive fixation loosening mechanism of fully threaded headless cannulated screw fixation for femoral neck fractures in young adults. J Orthop Surg Res. (2021) 16:234. 10.1186/s13018-021-02335-3 33785020 PMC8008647

[19] Huang S Zhang Y Zhang X Zhou C Li W Wang Y. Comparison of femoral neck system and three cannulated cancellous screws in the treatment of vertical femoral neck fractures: clinical observation and finite element analysis. Biomed Eng Online. (2023) 22:20. 10.1186/s12938-023-01083-1 36859241 PMC9979525

[20] Zhu Z Liao F. Optimal position and apex-subchondral bone distance of cannulated screws for internal fixation of femoral neck fracture: a radiological study. Journal of Orthopaedic Research: Official Publication of the Orthopaedic Research Society. (2023) 41:1546–54. 10.1002/jor.25503 36495052

[21] Stoffel K Zderic I Gras F Sommer C Eberli U Mueller D. Biomechanical evaluation of the femoral neck system in unstable pauwels III femoral neck fractures: a comparison with the dynamic hip screw and cannulated screws. J Orthop Trauma. (2017) 31:131–7. 10.1097/bot.0000000000000739 27755333

[22] Schopper C Zderic I Menze J Müller D Rocci M Knobe M. Higher stability and more predictive fixation with the femoral neck system versus hansson pins in femoral neck fractures pauwels II. J Orthop Translat. (2020) 24:88–95. 10.1016/j.jot.2020.06.002 32775200 PMC7387742

[23] Wu ZF Luo ZH Hu LC Luo YW. Efficacy of the femoral neck system in femoral neck fracture treatment in adults: a systematic review and meta-analysis. World J Clin Cases. (2022) 10:11454–65. 10.12998/wjcc.v10.i31.11454 36387785 PMC9649536

[24] Zhou Y Li Z Lao K Wang Z Zhang L Dai S. Femoral neck system vs. Cannulated screws on treating femoral neck fracture: a meta-analysis and system review. Front Surg. (2023) 10:1224559. 10.3389/fsurg.2023.1224559 37533744 PMC10390772

[25] Rajnish RK Srivastava A Rathod PM Haq RU Aggarwal S Kumar P. Does the femoral neck system provide better outcomes compared to cannulated screws fixation for the management of femoral neck fracture in young adults? A systematic review of literature and meta-analysis. J Orthop. (2022) 32:52–9. 10.1016/j.jor.2022.05.007 35601207 PMC9118353

[26] Yeoh S-C Wu W-T Peng C-H Yao T-K Chang C-M Liu K-L. Femoral neck system versus multiple cannulated screws for the fixation of pauwels classification type II femoral neck fractures in older female patients with low bone mass. BMC Musculoskelet Disord. (2024) 25:62. 10.1186/s12891-024-07179-6 38218794 PMC10787435

[27] Panteli M Rodham P Giannoudis PV. Biomechanical rationale for implant choices in femoral neck fracture fixation in the non-elderly. Injury. (2015) 46:445–52. 10.1016/j.injury.2014.12.031 25597514

[28] Xia Y Zhang W Zhang Z Wang J Yan L. Treatment of femoral neck fractures: sliding hip screw or cannulated screws? A meta-analysis. J Orthop Surg Res. (2021) 16:54. 10.1186/s13018-020-02189-1 33446230 PMC7807723

[29] Gao Y Ma T Chang X Jia P Li X Tang X. Femoral neck system (FNS) versus 4 cannulated compression screws (CCSs) in the treatment of young patients with pauwels type III femoral neck fracture: a retrospective comparative study. J Orthop Surg Res. (2025) 20:65. 10.1186/s13018-025-05461-4 39827144 PMC11742197

[30] Fan X Zhou Y Dai S Lao K Zhang Q Yu T. Bio-mechanical effects of femoral neck system versus cannulated screws on treating young patients with pauwels type III femoral neck fractures: a finite element analysis. BMC Musculoskelet Disord. (2024) 25:83. 10.1186/s12891-023-07110-5 38245678 PMC10799488

[31] Zielinski SM Keijsers NL Praet SFE Heetveld MJ Bhandari M Wilssens JP. Femoral neck shortening after internal fixation of a femoral neck fracture. Orthopedics. (2013) 36:e849–858. 10.3928/01477447-20130624-13 23823040

[32] Polat A Misir A Buyukkuscu MO Basilgan S Basar H. Factors associated with femoral neck shortening after closed or open reduction and screw fixation. Indian J Orthop. (2022) 56:303–11. 10.1007/s43465-021-00484-5 35140862 PMC8789974

[33] Duolikun D Chen Y Lou X-J Aierken A Zhang L Zheng L-P. Femoral neck system versus cannulated screws with a medial plate for the fixation of pauwels type III femoral neck fractures in young patients: a retrospective cohort study. BMC Musculoskelet Disord. (2025) 26:655. 10.1186/s12891-025-08910-7 40618107 PMC12228222

[34] Hasegawa K Takegami Y Tokutake K Nakashima H Mishima K Kobayashi K. Early versus delayed weight bearing after internal fixation for femoral neck fracture in younger adults: a multicenter retrospective study. Injury. (2024) 55:111292. 10.1016/j.injury.2023.111292 38157758

